# Lipid Accumulation and Insulin Resistance: Bridging Metabolic Dysfunction-Associated Fatty Liver Disease and Chronic Kidney Disease

**DOI:** 10.3390/ijms26146962

**Published:** 2025-07-20

**Authors:** Xinyi Cao, Na Wang, Min Yang, Chun Zhang

**Affiliations:** Department of Nephrology, Union Hospital, Tongji Medical College, Huazhong University of Science and Technology, Wuhan 430022, China; cxy19980410@126.com (X.C.); wnflora_2011@163.com (N.W.); mangmang1991@163.com (M.Y.)

**Keywords:** metabolic dysfunction-associated fatty liver disease, chronic kidney disease, oxidative stress, lipid, insulin resistance

## Abstract

Metabolic dysfunction-associated fatty liver disease (MAFLD), a recently proposed term to replace non-alcoholic fatty liver disease (NAFLD), emphasizes the critical role of metabolic dysfunction and applies broader diagnostic criteria. Diagnosis of MAFLD requires evidence of hepatic steatosis combined with obesity, type 2 diabetes mellitus, or other metabolic dysregulation conditions, all of which significantly elevate the risk of chronic kidney disease (CKD). This review discusses the pathological mechanisms of lipid accumulation and insulin resistance in MAFLD and CKD, highlighting their mechanistic connections. Specifically, ectopic fat accumulation triggered by metabolic reprogramming, oxidative stress and inflammation induced by energy overload, modified lipids, uremic toxins, and senescence, as well as insulin resistance pathways activated by pro-inflammatory factors and lipotoxic products, collectively exacerbate simultaneous hepatic and renal injury. Moreover, interactions among hyperinsulinemia, the sympathetic nervous system, the renin–angiotensin system (RAS), and altered adipokine and hepatokine profiles further amplify insulin resistance, ectopic lipid deposition, and systemic damage. Finally, the review explores potential therapeutic strategies targeting lipid metabolism, insulin sensitivity, and RAS activity, which offer promise for dual-organ protection and improved outcomes in both hepatic and renal systems.

## 1. Introduction

Metabolic dysfunction-associated fatty liver disease (MAFLD), previously termed non-alcoholic fatty liver disease (NAFLD), has emerged as a major cause of chronic liver disease and is accompanied by multi-system complications, including cardiovascular disease (CVD), diabetes mellitus, and chronic kidney disease (CKD) [1,2]. In a study of the Global Burden of Disease 2021 database, MAFLD was found to affect approximately 1.27 billion people worldwide, generated 48.35 million incident cases, and accounted for 3.67 million disability-adjusted life-years (DALYs), with the latter having risen by more than 60% since 1990 [3]. In 2020, international experts proposed renaming NAFLD as MAFLD to better reflect its underlying metabolic pathogenesis and improve patient management by recognizing extrahepatic manifestations. Unlike previous definitions that excluded significant alcohol consumption, the new diagnostic criteria emphasize metabolic dysfunction, specifically type 2 diabetes (T2D), overweight or obesity, and related metabolic disorders [4]. In 2023, a multisociety consensus further recommended renaming NAFLD to metabolic dysfunction-associated steatotic liver disease (MASLD) to explicitly underscore metabolic dysfunction as the primary etiological factor. Similar to the prior pathological characterization of NAFLD, MAFLD involves hepatic steatosis (defined as fat accumulation in over 5% of hepatocytes) and spans a clinical spectrum from benign steatosis to metabolic dysfunction-associated steatohepatitis (MASH), liver cirrhosis, and ultimately hepatocellular carcinoma [5,6]. Metabolic syndrome (MetS), the principal driver of MAFLD, encompasses obesity, hypertension, hyperglycemia, T2D, and dyslipidemia, all of which share insulin resistance as a central pathological mechanism [7]. Beyond metabolic-driven steatosis, chronic hepatitis C virus (HCV) infection remains a noteworthy differential diagnosis. The viral NS3/4A serine protease fosters hepatic steatosis, systemic insulin-resistance, and cryoglobulinaemic glomerulonephritis, thereby increasing the risk of CKD [8]. Cure rates > 95% with pangenotypic NS3/4A inhibitors and experimental RNA aptamers that bind HCV core or NS5B proteins—highlight the importance of ruling out occult HCV before attributing steatosis and renal injury to MAFLD/MASLD [9].

CKD is an irreversible, progressive disease characterized by persistent reduction in estimated glomerular filtration rate (eGFR) below 60 mL/min/1.73 m^2^, and/or abnormal urinary albumin-to-creatinine ratio (ACR, >30 mg/g) and/or overt proteinuria (>500 mg) lasting more than three months [10,11]. Pathologically, CKD manifests as renal inflammation and fibrosis, affecting tubular, glomerular, and interstitial structures [12]. CKD frequently coexists with obesity and ectopic fat deposition, promoting inflammation and structural damage in podocytes, mesangial cells, and proximal tubular cells [13]. Conversely, impaired renal function exacerbates lipid redistribution and dyslipidemia in CKD and end-stage renal disease (ESRD) [14]. Insulin resistance emerges as an initial metabolic alteration among CKD patients, even when eGFR remains within the normal range, and is particularly prevalent in ESRD. Insulin resistance accelerates cardiovascular and renal deterioration [15].

Globally, MAFLD and CKD affect approximately 30% and 9.5% of the population, respectively [16,17], posing substantial clinical and economic challenges. A systematic review and meta-analysis showed that the global prevalence of MAFLD was 50.7% in overweight and obese adults [18]. Additionally, a 15-year prospective study demonstrated that obesity severity is directly proportional to CKD incidence, with over 40% of severely obese participants developing CKD [19]. Recognized as a hepatic manifestation of MetS, MAFLD has a reciprocal relationship with obesity and insulin resistance. Importantly, MAFLD independently predicts CKD development, and liver fibrosis severity influences CKD prognosis [20]. Compared with traditionally defined NAFLD, MAFLD exhibits higher CKD prevalence, emphasizing critical role of metabolic dysfunction in connecting hepatic and renal pathologies [21]. The shared metabolic disorders, including obesity, dyslipidemia, and insulin resistance, likely serve as mechanistic links bridging MAFLD and CKD.

Despite acknowledging the mutual relationship between MAFLD and CKD, the precise molecular connections remain incompletely understood. This narrative review investigates the complexities of insulin resistance and lipid metabolism disturbances common to both diseases, examining their interrelationship and identifying potential therapeutic targets for clinical practice.

## 2. Pathology of Lipid Metabolic Disorders in MAFLD and CKD

### 2.1. Lipid Metabolism-Related Genes in MAFLD and CKD

Patatin-like phospholipase domain-containing protein 3 (PNPLA3), expressed in hepatocytes and human hepatic stellate cells (HSCs), encodes proteins that regulate intrahepatic triglyceride (TG) levels. A 2024 metabolomic study is the first to systematically compare differences in VLDL secretion and disease progression in three subtypes of MASLD; the PNPLA3 variant does not affect hepatic VLDL secretion, which is more common in metabotypes associated with CVD, whereas the transmembrane 6 superfamily member 2 (TM6SF2) variant is associated with reduced VLDL secretion [22].

Disruption of PNPLA3 ubiquitylation caused by gene variation leads to its accumulation in hepatic lipid droplets and potential hepatic steatosis [23]. The G allele of PNPLA3 rs738409 is the most significant and widespread variant influencing the progression of MAFLD, with the highest frequency in Central and South America (~50%) and the lowest in Africa (12%) [24]. This variant up-regulates the expression of lipid synthesis-related proteins such as cholesterol regulatory element-binding protein-1c (SREBP-1c) and carbohydrate response element-binding protein (ChREBP) and inhibits adipose TG lipase. PNPLA3 is expressed in podocytes and renal tubular cells, where the rs738409 variant contributes to renal lipotoxicity [25]. PNPLA3 rs738409 correlates with renal glomerular and tubular damage, including decreased glomerular filtration rate (GFR) and increased acute kidney injury (AKI) markers [26]

### 2.2. Lipid Overload, Steatosis, and Dyslipidemia of MAFLD

Increased de novo lipogenesis (DNL), increased fatty acid (FA) mobilization, and enhanced fatty acid β-oxidation (FAO) contribute to hepatic lipid accumulation and steatosis [7]. Elevated hepatic glucose and insulin independently enhance DNL by activating SREBP-1c and ChREBP, respectively, stimulating downstream lipogenic genes [6]. Citrate from an overloaded tricarboxylic acid (TCA) cycle shuttles into the cytoplasm, regenerating acetyl-CoA and exacerbating lipogenesis [27]. In the DNL process, the acetyl-CoA-to-malonyl-CoA conversion catalyzed by acetyl-CoA carboxylase (ACC) is enhanced by SREBP-1c up-regulation [28]. Malonyl-CoA inhibits carnitine palmitoyl transferase I (CPT-1), limiting FAO and exacerbating lipid accumulation [29]. Chronic lipid overload eventually disrupts FAO flexibility, causing endoplasmic reticulum (ER) stress, mitochondrial dysfunction and peroxisome reactions, and reactive oxygen species (ROS) production (e.g., O_2_•−, H_2_O_2_, and malondialdehyde (MDA)) [28]. In turn, excessive ROS aggravate mitochondrial dysfunction, establishing a positive feedback loop in hepatocyte apoptosis [30].

Peroxisome proliferator-activated receptor alpha (PPAR-α), predominantly expressed in the liver, regulates pathways of FAO and free FA (FFA) uptake, maintaining hepatic lipid and energy homeostasis. Under oxidative stress, ROS (H_2_O_2_) inhibit PPAR-α and CPT-1, reducing FAO rates [31]. Clinical studies reveal an inverse correlation between PPAR-α expression and hepatic steatosis severity [32]. Conversely, PPAR-γ, highly expressed in adipose tissue, promotes hepatic TG storage and DNL via SREBP-1c, exacerbating steatosis [33]. Energy depletion in the liver activates AMP-activated protein kinase (AMPK) and silent information regulator T1 (Sirt1), inhibiting the PPAR-γ/SREBP-1c pathway [34]. Meanwhile, PPAR-γ coactivator-1a (PGC-1α) can bind with PPAR-γ or PPAR-α to enhance CPT-1/FAO activity but is suppressed by insulin/SREBP-1c signaling and oxidative stress [35]. In adipose tissue, PPAR-γ augments lipid storage and adiponectin [36]. Adiponectin is negatively correlated with the degree of obesity, maintaining anti-inflammatory effects, FAO, and insulin sensitivity. Reduced adipose PPAR-γ promotes ectopic lipid accumulation, suggesting a complex, detrimental role in steatosis [37]. Although PPAR-γ acts complexly in the liver and adipose, its up-regulation in the liver and down-regulation in adipose tissue probably worsen steatosis.

Liver X receptor (LXR), as a cholesterol sensor of fat, glucose, and cholesterol metabolism, facilitates lipogenesis and fat storage by up-regulating SREBP-1c/CD36 to adapt to the energy excess of hepatic steatosis [38]. CD36 is an FA translocase that uptakes long-chain fatty acids (LCFAs), lipoproteins, modified lipids, and oxidized phospholipids (ox-PL), associated with apoptosis, angiogenesis, thrombosis, inflammation, and atherosclerosis. Clinical studies show that hepatic CD36 elevation drives fat accumulation in NAFLD patients, and it correlates with steatosis severity [39]. Farnesoid X receptor (FXR), highly expressed in the liver, ileum, and kidney, coordinates bile acid, lipid, and glucose homeostasis. FXR activation induces the transcription of PPAR-α/γ, CPT-1, and apolipoprotein C2 (Apo C2), while simultaneously repressing the LXR/SREBP-1c lipogenic axis [40]. During CKD progression, diminished expression of FAO enzymes, together with perturbations in key nuclear receptors (PPAR-α, PPAR-γ, and FXR), alters FA uptake machinery and downstream lipid–metabolic pathways, thereby accelerating tubulointerstitial fibrosis [41].

In MAFLD, excess FAs derived from DNL and adipose tissue lipolysis accumulate as intrahepatic TG droplets. Mobilization of these droplets markedly increases the secretion of TG-rich very-low-density lipoprotein (VLDL) into the circulation [42]. Within peripheral capillaries, lipoprotein lipase (LPL) and hepatic lipase hydrolyze VLDL and chylomicrons, releasing FFAs; the residual particles are remodeled into intermediate density lipoprotein (IDL), cholesterol-rich low-density lipoprotein (LDL-c), and chylomicron remnants (CM). Concurrently, Apo A1, cholesterol, and phospholipids assemble into nascent high-density lipoprotein (HDL), which lecithin-cholesteryl acyl-transferase (LCAT) converts to mature cholesteryl-ester (CE)-rich HDL (HDL-c). Cholesteryl-ester transfer protein (CETP) then exchanges HDL-c for TG in LDL, generating TG-enriched HDL (HDL-TG) and LDL-c destined for hepatic clearance. MAFLD is characterized by heightened CETP activity, over-production of VLDL, accumulation of small dense LDL (sdLDL) particles, and depressed HDL-c concentrations—an atherogenic profile that predisposes to cardiovascular disease [43]. Angiopoietin-like proteins (ANGPTLs), frequently upregulated in MAFLD, further inhibit LPL and impair peripheral FA uptake [44].

### 2.3. Dyslipidemia and Adipose Ectogenesis in CKD

Dyslipidemia emerges early in CKD, distinguished from the MAFLD pattern by reduced activity of LPL and hepatic lipase, down-regulation of VLDL receptors (VLDLR) in muscle and adipose tissue, and diminished hepatic LDL-receptor-related protein 1 (LRP-1) [45,46]. Advancing CKD also elevates proprotein convertase subtilisin/kexin type-9 (PCSK9), which suppresses LDL-receptor (LDLR) expression [47]. Impaired lipoprotein remodeling hampers the transition from pre-β-HDL to mature HDL; uremic retention of apolipoprotein C-III (Apo C-III) and pre-β-HDL further inhibits LPL-mediated TG clearance, an effect amplified by secondary hyperparathyroidism [48,49,50]. In ESRD, hemodialysis exacerbates LPL depletion and lowers Apo A1 and HDL-c levels [51]. Overall, CKD is typified by low HDL-c, abundant sdLDL, and progressively rising CETP activity with declining eGFR, while LCAT activity falls, culminating in extremely low HDL-c and HDL-TG particles [47].

**Figure 1:**
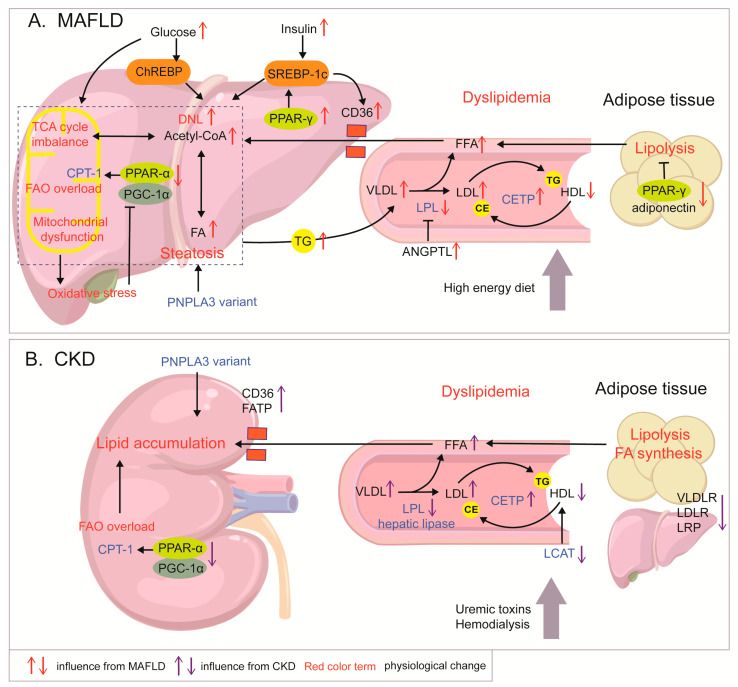
Mechanisms of lipid metabolism abnormalities in MAFLD and CKD. (**A**) In MAFLD, DNL enhanced by elevated insulin and glucose levels, FAO overload, and increased FA influx leads to lipid accumulation and steatosis. Excessive energy contributes to TCA mitochondrial dysfunction and ROS production. In turn, oxidative stress inhibits the PGC-1α/CPT-1/FAO axis. PPAR-γ shows opposite expression patterns in the liver and adipose tissue, enhancing hepatic DNL and adipocyte lipolysis, respectively, and exacerbating fat accumulation in MALFD. Increased levels of TG-containing VLDL are released into circulation, together with decreased HDL-c and increased LDL-c levels, which are regulated by increased CETP and inhibited LPL, forming dyslipidemia. (**B**) In CKD, abnormality of enzymes including LPL, LCAT, and CETP and dysregulated expression of VLDLR, LDLR, and LRP contribute to CKD dyslipidemia, characterized by increased LDL-c levels, elevated VLDL levels, and impaired HDL. Abnormal lipid enzymes in CKD are also related to uremic toxins and dialysis status. Down-regulation of the PGC-1α/CPT-1 pathway in the renal tubules and up-regulation of transporters such as CD36 cause lipid accumulation in the kidneys.

Although the kidney is intrinsically a “lean” organ, CKD triggers ectopic lipid accumulation. In the kidneys, proximal tubular epithelial cells (PTECs) are the major oxygen-consuming cell type and exert FAO activities regulated by related genes such as PPARs and CPT-1, in which PPAR-α is highly expressed [52]. CKD suppresses the FAO axis (PPAR-α, PGC-1α, and CPT-1) and AMPK, and FA synthesis is enhanced in PTECs [52]. Moreover, FA synthesis and the expression of lipid synthesis-related enzymes are augmented in the adipose tissue of rats suffering from chronic renal failure [53]. In addition to suppressed intrarenal metabolism, fatty acid transport protein (FATP), fatty acid-binding protein (FABP), and scavenger CD36 are widely expressed in the kidneys and can facilitate lipid accumulation. Membrane transporters (CD36 and FATP2) are upregulated after kidney injury or exposure to uremic milieu, leading to renal lipid accumulation [54]. The pathological mechanisms of lipid abnormality in MAFLD and CKD are shown in Figure 1.

## 3. Lipid Burden Linking MAFLD and CKD

### 3.1. Abdominal/Visceral Obesity

Abdominal obesity correlates closely with both hepatic and renal lipid deposition. Recent clinical studies have verified that prolonged sitting, consumption of sugar-sweetened beverages (SSBs), smoking, hormone changes, etc., are all factors that enhance visceral adipose tissue (VAT), and these factors are further stratified by age, gender, and region [55,56,57].

In a National Health and Nutrition Examination Survey III cohort study, the coexistence of MAFLD and abdominal obesity raised the multivariable adjusted odds of CKD by 84%, and central fat indices mediated approximately 70% of the MAFLD-to-CKD pathway, whereas BMI explained only 33% [58]. It also showed that post-menopausal women had higher incidence of MAFLD and steeper eGFR decline [58]. Loss of hepatic estrogen/estrogen receptor-α signaling curtails FAO, enhances DNL, and compromises insulin pathways [59]. However, before this point in time, visceral fat accumulation and MAFLD prevalence is generally higher in men than that in women [3]. Estrogen up-regulates PNPLA3 to promote TG storage in subcutaneous adipose tissue (SAT), whereas androgen increases the VAT/SAT ratio; sex hormones also influence eating style, with men preferring fats and women preferring carbohydrates [60]. Smoking is an important factor contributing to the higher incidence of MAFLD in men aged 19–39, and nicotine activation of the renin–angiotensin system (RAS) is even more detrimental to CKD prevention [61].

### 3.2. Obesity-Related Inflammation

The liver acts as an active secretory organ rather than a passive reservoir. Hepatocellular lipid droplet overload provokes ER stress and reactive oxygen species (ROS) generation, activating Kupffer cells and the nuclear factor kappa B (NF-κβ) and c-Jun N-terminal kinase (JNK) pathway, releasing inflammatory factors and chemokines. Notably, senescent hepatocytes accumulate with age and MASLD severity; they lose β-oxidation capacity and secrete senescence-associated secretory phenotype (SASP) cytokines rich in IL-6, IL-1β, and monocyte chemoattractant protein 1 (MCP-1), which trigger NOD-like receptor family pyrin domain containing 3 (NLRP3) systemically [62]. Consequently, MASLD augments the secretion of pro-inflammatory factors and hepatokines such as fetuin-A and ANGPTLs remain chronically elevated, exacerbating fat redistribution and peripheral insulin resistance [63].

In adipose tissue, excess lipids and lipotoxic intermediates sustain macrophage-driven inflammation, enhance lipolysis, and induce insulin resistance [37]. Activation of JNK and NF-κB in adipocytes and adipose tissue macrophages (ATM) promotes the secretion of TNF-α, IL-6, IL-1β, angiotensin II, and leptin [6]. These cytokines worsen metabolic dysfunction in the liver and adipose tissue and have been implicated in podocyte effacement and increased glomerular permeability [64]. Moreover, IL-6 synergizes with angiotensin II to amplify intrarenal RAS signaling in proximal tubular epithelial cells (PTECs) [65]. The oxidative stress, metabolic dysfunction, and ischemia hits also contribute to the SASP of PTECs, which inhibits renal regeneration and exacerbates the process of fibrosis [66].

### 3.3. Lipoproteins and Lipotoxic Products

MAFLD derived dyslipidemia is characterized by increased levels of VLDL, LDL-c, and sdLDL, and decreased levels of HDL, but CKD is dominated by metabolic enzyme dysfunction, LDL-c excess, and HDL immaturity. When circulating lipid levels surpass the storage capacity of adipose tissue, lipids become deposited in multiple organs besides adipose tissue (e.g., liver, kidney, and muscle), a phenomenon termed “ectopic lipid deposition” [67]. Filtered FFAs and oxidized LDL (ox-LDL) are re-absorbed by PTECs, and lipid deposition in diverse renal cell types precipitates nephrotoxicity. In the liver, dysfunctional HDL and atherogenic lipoproteins accelerate lipid accumulation and atherosclerosis—major determinants of NASH-related mortality [68]. For the kidney, staged reduction of organic anion transporter (OAT1/3) and HDL-receptor-mediated uptake in CKD makes the excretion of protein-bound/particle-bound lipid metabolites more difficult. Circulating lipoproteins, including VLDL and LDL, heighten the permeability of the glomerular basement membrane by binding to polyanionic glycosaminoglycans; the filtered lipoproteins amass within mesangial cells to stimulate cell proliferation and production of the basement membrane substance, culminating in glomerular and tubulointerstitial disease [69].

ROS-mediated oxidation converts LDL into ox-LDL, which amplifies pro-inflammatory signaling and oxidative stress [70]. Ox-LDL facilitates monocyte recruitment to the kidney, enhances macrophage adhesion to the glomerular endothelium, and promotes macrophage-to-foam-cell transformation via CD36 [71,72]. In the dyslipidemia mouse model, podocyte injury was found to drive foam cell accumulation in capillaries, ultimately leading to segmental glomerulosclerosis [73]. Ox-LDL-CD36/Toll-like receptor (TLR4)-dependent endocytosis triggers ROS, NLRP3 activation and cuproptosis in renal tubules, accelerating necrosis and fibrosis [74], and stimulates TGF-β1–driven fibrogenesis in mesangial cells and PTECs [75,76]. HDL normally restrains ROS production and LDL oxidation; its quantitative and functional decline therefore intensifies atherosclerosis and CKD progression [77]. It can be seen that the metabolic communication between MAFLD and CKD tends to be a “liver-generated and kidney-retained” relationship, but the impact from CKD should not be ignored. Ox-LDL formation is also accelerated in CKD, partly owing to myeloperoxidase (MPO) excess [78]. Similarly, ox-LDL induces intrahepatic ROS surge and mitochondrial dysfunction. In early stages of atherosclerosis, Apo B-containing ox-LDL is efficiently phagocytosed by Kupffer cells, triggering intracellular inflammatory transcription and short-term induction of intrahepatic cholesterol/TG accumulation [79]. Ox-LDL can directly up-regulates NF-κβ/TGF-β1 signaling in HSC to drive collagen 1A1 deposition and fibrosis [80]. A 2025 study found that ox-LDL-induced CD36 upregulation in double-negative regulatory T cells precipitates ferroptosis and disrupts hepatic immune homeostasis in MASLD [81]. Ox-LDL elicits an immune cell response and fibrotic signaling activation in both the liver and kidneys, which is pathologically significant in connecting liver-kidney circuit.

### 3.4. Modified Lipids as a New Class of Uremic Toxins

In CKD, reduced antioxidant defenses and impaired renal excretion favor the accumulation of lipid-derived uremic toxins, including 3-carboxy-4-methyl-5-propyl-2-furanpropanoic acid (CMPF), advanced lipoxidation end-products (ALEs), and ox-PL [82]. Levels of ALEs and ox-PL/ox-LDL also rise in steatohepatitis [28,83]. Although not much literature on CMPF and fatty liver exists, a clinical study of NAFLD patients showed that elevated CMPF is negatively correlated with lipid metabolizing ability, with the potential to predict disease progression [84]. ALEs, such as MDA and 4-hydroxy-2-nonenal (4-HNE), the end products of poly unsaturated fatty acid (PUFA) peroxidation, are initiated by ROS attack on membrane PUFAs. They serve as both oxidative-stress markers and endothelial toxins [85]. 4-HNE-modified LDL, for instance, accelerates foam-cell formation and endothelial permeability [82]. Lipid-derived uremic toxins and lipotoxic products predispose to vascular problems, amplified inflammation, and metabolic reprogramming, which are the mediators linking CKD to MAFLD progression. Antioxidant and anti-inflammatory dysfunction contributes to increased ROS and elevated ALEs, resulting in increased pro-inflammatory factors including MCP-1, IL-6, IL-1β, and TNF-α. These cytokines, acting as pro-insulin resistance mediators, contribute to increased FFA release, reduced glucose uptake, and energy influx to the liver [27]. CC chemokine receptor 2 (CCR-2) expression is elevated in the liver in MAFLD, and MCP-1 binding to CCR2 causes more monocyte and macrophage infiltration in the liver [86]. ALE binding to the receptor for advanced glycation end products (RAGE) simultaneously activates NF-κB and TGF-β1 in hepatocytes, Kupffer cells, and HSCs, fueling an inflammatory-fibrotic circuit and dysregulated iron homeostasis [87]. Similar to ox-LDL, Ox-PL binding to TLR2/TLR4 causes a burst of ROS. As demonstrated by Sun et al., ox-PL suppressed PGC-1α-dependent FAO and promoted mitochondrial dysfunction and hepatic fibrosis, a process reversible with the E06 neutralizing antibody [83]. Oxidized lipoproteins, phospholipids, and inflammatory factors are among the damage-associated molecular patterns (DAMPs), and sensors such as RAGE, CD36, and TLR are effectors for DAMPs. Blocking this signaling process, antioxidant or neutralizing antibodies are promising therapeutic directions.

### 3.5. Lipoprotein Recepters and Effectors

In CKD, hepatic LRP-1 and LDLR and peripheral tissue VLDLR are down-regulated; LRP-1 and LDLR also decline in MAFLD, whereas VLDLR is markedly up-regulated in advanced steatosis [88]. LRP-1 loss leads to CM and IDL retention and exacerbates hepatic TG/cholesterol accumulation, thereby worsening steatohepatitis under high-fat dietary (HFD) intake [89]. Decreased VLDLR/LPL impairs peripheral VLDL-TG uptake, contributing to CKD-associated sarcopenia and insulin resistance [90]. The surplus VLDL-TG is redirected to the kidney, liver, and vasculature and, together with hepatic VLDLR changes, facilitates excessive intrahepatic lipid recycling.

CD36 expression is markedly elevated in circulating monocytes/macrophages and renal tissue of dialysis patients [54]. In podocytes, CD36-mediated palmitate uptake induces ROS production, inflammation, and apoptosis [91]. Pharmacological or genetic CD36 inhibition prevents renal fibrosis in experimental models, positioning CD36 as a therapeutic target [92]. Obesity-related podocyte detachment increases glomerular permeability, while sodium–glucose cotransporter 2 (SGLT-2)-mediated glucose hyper-reabsorption exacerbates tubular injury [93]. In the liver, CD36 drives lipotoxic uptake, insulin resistance and steatosis [94]. Monocytes are recruited to tissues at the onset of liver injury; an increase in non-classical monocytes increases the risk of NAFLD [95]. CD36 is highly expressed in monocytes/macrophages infiltrating fibrotic livers [96], suggesting that highly CD36-expressing monocytes recruited to the liver may exacerbate hepatic fibrosis. It is verified that ox-LDL up-regulates the expression of macrophage CD36 by the long non-coding RNA MALAT1 (LncMALAT1) [97]. Ox-LDL also accentuates hepatocyte LXR/CD36 signaling, reinforcing hepatic lipotoxicity [98].

Due to the widespread expression of CD36, its role in lipid metabolic reprogramming is potentially considered as a liver–kidney therapy. Vivo and in vitro studies have found that some natural CD36 inhibitors prevent or mitigate fat accumulation in NAFLD, such as quercetin and alisol B [99]. Apo AI-mimetic peptide 5A competitively occupies CD36 and promotes LDL-c efflux. In a CKD mice model, the 5A peptide inhibited NLRP3 activation, macrophage infiltration, and presented remission of albuminuria and interstitial fibrosis [100]. However, natural CD36 inhibitors have multiple targets of action, and mimetic short peptides may cause excess serum LDL-c and immune cell dysregulation. Their safety and clinical feasibility need to be confirmed in long-term studies.

**Figure 2:**
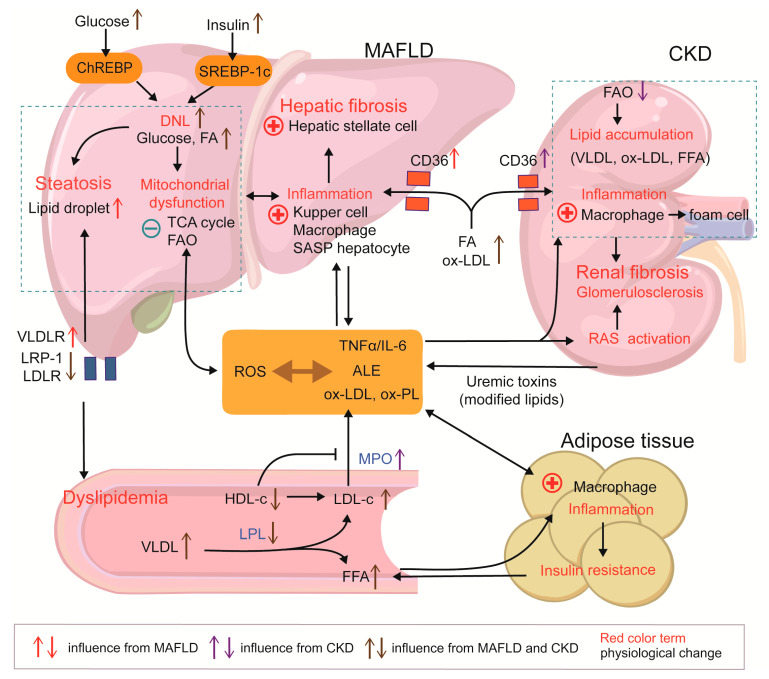
Metabolic dysfunction linking MAFLD and CKD. (MAFLD) and chronic kidney disease (CKD) share a dyslipidemic signature, including decreased HDL-C, elevated LDL-C and VLDL, and altered lipoprotein receptor profiles. Hepatic lipid overload precipitates mitochondrial dysfunction and ROS generation, whereas impaired toxin clearance in renal failure magnifies this oxidative milieu. ROS drive the formation of ALE, ox-LDL, ox-PL, and other pro-inflammatory danger-associated molecular patterns, fostering systemic multi-organ inflammation. Adipose inflammation aggravates insulin resistance and liberates FFAs, accelerating lipid flux to both the liver and kidney, which is ectopic fat accumulation. In the liver, ALEs and CD36-mediated ox-LDL uptake activate resident immune cells and trigger HSC-driven fibrosis. In the kidney, deposition of ox-LDL, FFAs, and other lipoproteins induces macrophage foam-cell formation and promotes glomerulosclerosis and fibrosis.

To conclude, lipid mobilization and redistribution are associated with fat inflammation, circulating lipotoxins, and related receptor alterations in peripheral tissues at the onset of both diseases, and this is particularly obvious when MAFLD is accompanied by CKD. MAFLD, as one of the MetS phenotypes, is more likely to be a predisposing factor for CKD, but CKD also affects lipid metabolism and leads to hepatic injury (Figure 2).

## 4. Pathology of Insulin Resistance in MAFLD and CKD

### 4.1. Lipotoxicity, Inflammation, and Insulin Resistance in MAFLD

**Figure 3:**
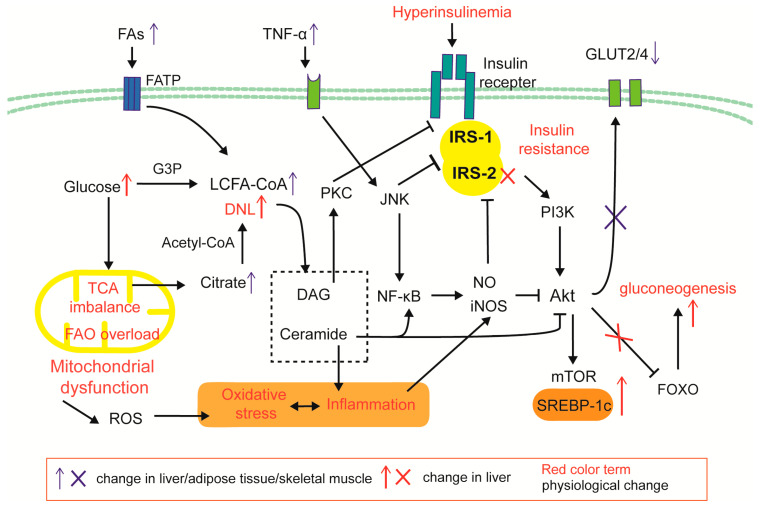
Cellular mechanism of hepatic and peripheral insulin resistance. Under obesity and systemic inflammation, FA input and cytokine stimulation is increased. FAO/TCA imbalance and mitochondrial dysfunction causes an increase in ROS and lipotoxic products (DAGs and ceramides) of DNL. The inflammatory factor TNF-α activates the NF-κβ/JNK and iNOS-NO pathways, impairing the IRS/Akt/GLUT2/4 insulin signaling pathway; DAG/PKC signals inhibit the insulin receptor, and ceramide induces the iNOS inflammatory pathway and inhibits Akt. Hepatic insulin resistance is selective: Inactivation of the IRS2/AKT/FOXO signaling pathway leads to a decrease in insulin’s ability to inhibit glucose metabolism; due to differences in the intrahepatic expression distribution of IRS-1 and IRS-2, the IRS-1/Akt/mTOR pathway is less affected by PKC signaling, thereby enhancing SREBP-1c/DNL.

Adipose tissue is the principal site of systemic insulin resistance, functioning as an FFA pump [101]. During the early stages of obesity or high-fat feeding, adipocyte hypertrophy precipitates local hypoxia, fibrosis, and macrophage infiltration. The ensuing release of IL-6 and TNF-α accelerates lipolysis and aggravates insulin resistance, further amplifying FFA efflux. In skeletal muscle, ectopic lipid deposition impairs glycogen synthesis and glucose uptake, redirecting energy substrates toward hepatic lipogenesis [7]. Hepatic insulin resistance exists independently and secondarily to insulin resistance in adipose tissue and skeletal muscle [102]. Pancreatic lipid overload initially augments insulin secretion, but chronic exposure to excess glucose and FFAs induces oxidative stress, inflammation and ultimately β-cell dysfunction and apoptosis [102,103]. The mechanisms by which insulin resistance occurs in the liver and peripheral tissues are described below and presented in Figure 3.

Excess nutrient supply provokes mitochondrial dysfunction and endoplasmic reticulum (ER) stress, increasing ROS production and activating TLR, NF-κB and JNK signaling [102]. JNK, upregulated in insulin-responsive tissues including the liver, adipose tissue, and skeletal muscle of T2D patients, phosphorylates insulin receptor substrate 1/2 (IRS-1/2) on serine residues, thereby disrupting downstream phosphatidylinositol 3-kinase (PI3K)/serine threonine kinase (Akt) signaling [104]. IRS/PI3K/Akt is a core pathway of insulin signaling that inhibits hepatic gluconeogenesis, increases glycogen levels, and promotes glucose uptake through the translocation of glucose transporters 2/4 (GLUT 2/4; GLUT2 in liver; GLUT4 in adipocytes and skeletal muscle). In NAFLD models, TNF-α neutralization alleviates JNK-dependent IRS-1 phosphorylation and JNK/SREBP-1c-driven IRS-2 down-regulation, restoring hepatic insulin sensitivity [105]. Moreover, FFA-stimulated JNK activation similarly compromises GLUT4 translocation and glucose uptake in adipocytes and muscle cells [106,107]. In response to inflammation and oxidative stress, inducible nitric-oxide synthase (iNOS), transcriptionally upregulated by NF-κB and JNK, amplifies inflammation by generating nitric-oxide (NO)-derived reactive species (RNS), such as peroxynitrite (ONOO^−^), nitroxyl anion (NO^−^), and dinitrogen trioxide (N_2_O_3_) [108]. iNOS was found to be elevated in both Kupffer cells and hepatocytes in an NAFLD and obesity model; it generates NO/RNS to promote the S-nitrosylation of IRS-1 and the down-regulation of IRS-2 in hepatocytes [108,109]. In skeletal muscle, lipopolysaccharide/palmitate-induced iNOS promotes the nitration of insulin receptor tyrosine kinase (INSRβ), IRS-1, and Akt, further weakening insulin signaling [110]. Blockade of INSR/IRS/Akt/GLUT4 pathway and iNOS/RNS elevation also occurs in hypertrophic and stressed adipocytes [111]. Thus, JNK and iNOS constitute mutually reinforcing inflammatory nodes that impair insulin action across multiple tissues.

In hepatocytes, non-essential fatty acids (NEFAs), especially long-chain fatty acids (LCFAs) and their lipotoxic products, such as diacylglycerols (DAGs) and ceramide, are significantly enriched, which exacerbate hepatic insulin resistance. DAGs and lipotoxic products also influence insulin resistance in muscle. During DNL, glycerol-3-phosphate (G3P, from glycolysis) esterifies LCFA-CoA to generate lysophosphatidic acid, a DAG precursor. Metabolic overload can shift energy flow from the pathways of FAO and glycolysis to DNL and gluconeogenesis [27]. Excessive glucose and LCFA-CoA can be transformed into G3P to promote DAG production [37]. DAGs activate protein kinase C (PKC) to inhibit INSRβ and insulin-related IRS-2 phosphorylation. DAG activation of PKCs inhibits INSRβ and IRS-2 phosphorylation, relieving forkhead box protein O (FOXO) inactivation and up-regulating gluconeogenesis-related enzymes [112]. As IRS-1 is less affected by PKC, the IRS-1/mTOR and the DAG/PKC/mTOR pathway can still up-regulate SREBP-1, while glucose-induced ChREBP activation remains insulin-independent [113]. Different from the liver, adipose tissue has lower glucose uptake, insufficient glucose-ChREBP activation, and reduced DNL.

In the Golgi apparatus and ER, LCFA-CoA (e.g., palmityl-CoA) and sphingosine are esterized into ceramide. FA-CD36-mediated sphingomyelin hydrolysis will increase ceramide levels [37]. Ceramide is verified to impede insulin signaling in vitro, and in one study, it inhibited glucose uptake, promoted lipid storage, and induced hepatic steatosis in mice [114]. Specifically, ceramide can inhibit Akt and activate the NF-κβ signaling pathway, thus inducing iNOS expression, inflammation and impairing insulin signaling [36,102]. IRS1 is predominantly expressed in the hepatic perivenous zone responsible for lipogenesis, whereas IRS-2 expression is concentrated in the hepatic periportal zone responsible for gluconeogenesis. IRS-2 is susceptible to suppression by hyperinsulinemia, which may be achieved through inactivated FOXO and activated SREBP-1c, whereas IRS-1 in the lipogenesis zone is relatively preserved [7]. Consequently, hepatic insulin resistance is “selective”: glucose uptake and glycogen synthesis decline, yet gluconeogenesis and lipogenesis remain inappropriately active.

### 4.2. Insulin Resistance in CKD

Independent insulin resistance frequently accompanies CKD, emerging in early stages and becoming nearly universal in ESRD [15]. Skeletal muscle is the primary site of CKD-derived insulin resistance; the rodents with subtotal nephrectomy exhibited marked GLUT4 depletion [115]. Similar to MAFLD, CKD patients exhibit excess FFAs and abnormal levels of adipokines (e.g., adiponectin, leptin, resistin, and omentin) in their circulation, leading to systemic insulin resistance in peripheral tissue [15]. Insulin resistance among CKD patients is associated with reduced adiponectin levels, and a decline in adiponectin levels is associated with the risk of CVD [116]. Leptin, normally cleared by kidneys, accumulates as GFR falls; chronic hyperleptinaemia is linked to sympathetic over-activity and insulin resistance in uremic patients [117]. Though slight increases in leptin are beneficial for fat metabolism, chronic hyperleptinemia triggers insulin resistance in uremic patients [15]. Uremic toxins, including carbamylated proteins and protein-bound toxins, generate ROS and impair insulin signaling in peripheral tissues [118].

In haemodialysis patients, insulin resistance correlates with deficient erythropoietin (EPO) and 1,25 dihydroxycholecalciferol [1,25(OH)2D3]. Reduced EPO secretion, a consequence of renal insufficiency, is a primary contributor to renal anemia development [119]. EPO improves insulin sensitivity by improving anemia and diminishing TG levels [120]. Anemia may have induced insulin resistance through chronic tissue hypoxia and metabolic stress. Although a direct causal link between renal anemia and insulin resistance remains unproven, chronic hypoxia and metabolic stress are plausible mediators. As kidney disease advances to its end stage, a deficiency in calcium and 1,25(OH)2D3 coupled with elevated phosphate levels instigates the onset of secondary hyperparathyroidism, causing a compensatory elevation in parathyroid hormone (PTH). Excessive PTH concentrations impair the insulin secretion of pancreatic β-cells via calcium-dependent mechanisms, obstructing glucose uptake [121]. Dialysis patients deficient in 1,25(OH)2D3 exhibited glucose intolerance in one study [122]. Researchers found that 1,25(OH)2D3 treatment and vitamin D receptor agonist (VDRA) attenuated fibrosis and albuminuria in CKD patients by inactivating RAS, though vitamin D application has not been found to delay the progression of CKD [123].

## 5. Insulin Resistance Linking NAFLD and CKD

### 5.1. The Linkage Between Ectopic Lipid Accumulation and Insulin Resistance

In CKD, insulin resistance typically manifests first in the skeletal muscle and subsequently involves adipose tissue and the liver [15]. Diminished peripheral glucose uptake, enhanced lipolysis, and compensatory hepatic energy production drive this metabolic phenotype. A study has shown that advanced CKD contributes to the up-regulation of SREBP-1/ChREBP-mediated DNL-associated enzymes and the depression of CPT-1-mediated FAO activity in the liver, accompanied by hepatic fat accumulation [124]. The role of CKD in metabolic reprogramming of the liver possibly reflects an insulin-resistant milieu, adipokine dysregulation, and chronic inflammation. Insulin resistance and CKD together potentiate CETP activity and the fractional catabolic rate of Apo A1, culminating in immature HDL particles [125]. Declining HDL-c prompts a compensatory rise in VLDL-TG and an elevated remnant cholesterol/HDL-c ratio. The immaturity of HDL impedes the reverse transport of cholesterol from peripheral tissues to the liver and affects the clearance of free cholesterol in the liver, thereby triggering ER stress and TG synthesis and promoting the progression of NAFLD and atherosclerosis [126]. AMPK-driven FAO is weakened and LXR/SREBP-1c is activated due to cholesterol accumulation and HDL exhaustion [127]. Consequently, insulin resistance constitutes an independent risk factor for NAFLD and accelerates the transition to fibrotic NASH [128].

In MAFLD, insulin resistance manifests as a hepatic “lipogenic-gluconeogenic phenotype” and a peripheral “glucose-FFA-release phenotype,” together establishing a supply-and-storage circuit at an early stage. Intrahepatic DNL inversely correlates with systemic insulin sensitivity and fuels TG storage [129]. Dysfunction of the IRS/Akt/FOXO pathway due to selective hepatic insulin resistance results in a loss of inhibition of Apo B lipidation, contributing to a significant increase in the secretion of Apo B-containing lipoproteins, such as VLDL [130]. The resulting VLDL-TG, along with FFAs and glucose derived from peripheral insulin resistance, fosters renal lipotoxicity and glucotoxicity. Lipoproteins stimulate mesangial cell proliferation and extracellular-matrix (ECM) production [69]. NEFA deposition in podocytes and proximal tubular epithelial cells (PTECs) activates mTOR, promoting gluconeogenesis, proteinuria, and tubular injury [13]. Su et al. found that palmitate inhibited PPAR-γ expression to reduce the renal levels of insulin-degrading enzyme (IDE), which participated in the clearance of insulin and insulin sensitivity [131]. Advanced glycation end-products (AGEs) engage RAGE on PTECs, activating metalloproteinase-2 (MMP-2) and ROS and accelerating diabetic nephropathy [132]. DAG-PKCβ activation is also involved in renal vascular permeability, immune cell adhesion, and cytokine activation [133]. We speculate that insulin resistance contributes to lipid accumulation in the kidneys and liver (Figure 3).

### 5.2. Adipokines and Hepatokines

MAFLD is characterized by elevated leptin and reduced adiponectin levels secondary to obesity and inflammation; early CKD shows an analogous pattern owing to diminished renal clearance, although adiponectin levels rise in later CKD stages. Adiponectin enhances insulin sensitivity and FAO via the AMPK/PPAR-α pathway; its decline promotes HSC activation and ROS generation [134]. Decreases in adiponectin and increases in leptin levels can lead to podocyte injury and albuminuria [135]. A slight increase in leptin promotes FAO, but an excessive increase promotes ROS production and enhances fibrosis signaling. Leptin excess induces podocyte damage, albuminuria, and hypertension through TGF-β1 signaling [136] and can magnify NAFLD by eliciting pyroptotic hepatocyte death via CD8+ T cell activation [137]. A 2023 study of 575 NAFLD patients found that the adiponectin–leptin ratio (ALR) could predict disease severity independently of insulin resistance [138]. There are currently no such cohort studies for CKD, but it is evident that a high adiponectin-leptin ratio is detrimental to glomerular function. CKD and MAFLD can drive each other through this adipokine abnormality, with or without inducing insulin resistance. Fetuin-A levels rise in both MAFLD and obesity-related CKD, potentiating lipid-driven inflammation, fibrogenesis, and adiponectin suppression; thus, Fetuin-A-mediated AMPK inhibition may represent a common pathological node [134].

Fibroblast growth factor 21 (FGF-21) is mainly secreted in the liver, but other organs can also be its producers and targets. FGF-21, the hepatokine mediated by PPAR-α activation, is compensatorily up-regulated in MAFLD to maintain energy homeostasis [63]. FGF-21 alleviates lipid metabolism through the inhibition of SREBP-1c and increases in the expression of PGC-1α in the liver and maintains insulin sensitivity through activation of the PI3K/Akt/GLUT4 signaling pathway, adiponectin up-regulation, and direct protection of pancreatic β-cells [63,139]. FGF-21 also alleviates renal inflammatory infiltration and lipotoxicity, but FGF-21-induced hypertension is noteworthy [140]. Levels of Β-Klotho (KLB), the anti-aging molecule that mediates the signaling of FGF receptor 1c (FGFR-1c), decline with age, CKD progression, and inflammation, contributing to FGF-21 resistance [141]. FGF-21 increases with aging and severity of MAFLD and metabolic dysfunction; although it has a protective effect, impaired signaling still renders FGF-21 ineffective [142]. As a protective factor linking MAFLD and CKD, FGF-21 can serve as a common therapeutic target.

Insulin-like growth factor 1 (IGF-1) modulates insulin sensitivity and protein synthesis. NAFLD displays reduced IGF-1 levels and elevated levels of IGF-binding proteins (IGFBPs), whereas advanced fibrosis is associated with further IGF-1 decline and heightened CKD risk [139]. IGFBP is detrimental to IGF-1’s effect, and its up-regulation contributes to IGF-1 resistance. A clinical study of adults with NAFLD demonstrated an increased risk of progression to CKD in the group with a high-risk of fibrosis, which was accompanied by a decrease in IGF-1 [143]. Moreover, CKD presents elevated IGFBP, growth hormone (GH)/IGF-1 resistance, and IGF-1 elevation [144]. Normal levels of IGF-1 help maintain eGFR, promote repair, and suppress inflammation; excessive IGF-1 mediates renal ECM accumulation, cell proliferation and fibrosis [145]. Both diseases show biphasic dysregulation—initial deficiency followed by relative resistance—underscoring the need for time-specific therapy.

### 5.3. SNS-RAS-ROS Axis

Systemic insulin resistance impairs PI3K-mediated NO production, favoring vasoconstriction and glomerular hyperfiltration [146]. Hyperinsulinemia promotes SNS hyperactivity and sodium retention, which is verified in obesity, T2D, and renal failure [147]. SNS activation causes the release of angiotensin–aldosterone and norepinephrine (NE) to promote renal vasoconstriction and hypertension. Angiotensin II and ROS reinforce one another and together with hyperglycemia activate epidermal growth factor receptor (EGFR)/Src/TGF-β1 signaling, driving ECM deposition and glomerulosclerosis [148]. Insulin also up-regulates vascular AT1-receptor expression, sensitizing tissues to angiotensin II and promoting renal vasoconstriction and sodium–water retention [149,150]. Under hyperinsulinemia and obesity conditions, RAS activation and enhanced sodium–glucose absorption in the proximal tubules result in hyperfiltration, albuminuria, and potential glomerulosclerosis [13]. Under inflammatory conditions, infiltrating macrophages release IL-6 to mediate the overexpression of cell adhesion molecules 1 (CAM-1) and AT-1 receptors in the endothelium [151]. AT-1 receptors facilitate renal vascular damage, and CAM-1 aids in the recruitment of macrophages to the vascular endothelium [152].

**Figure 4:**
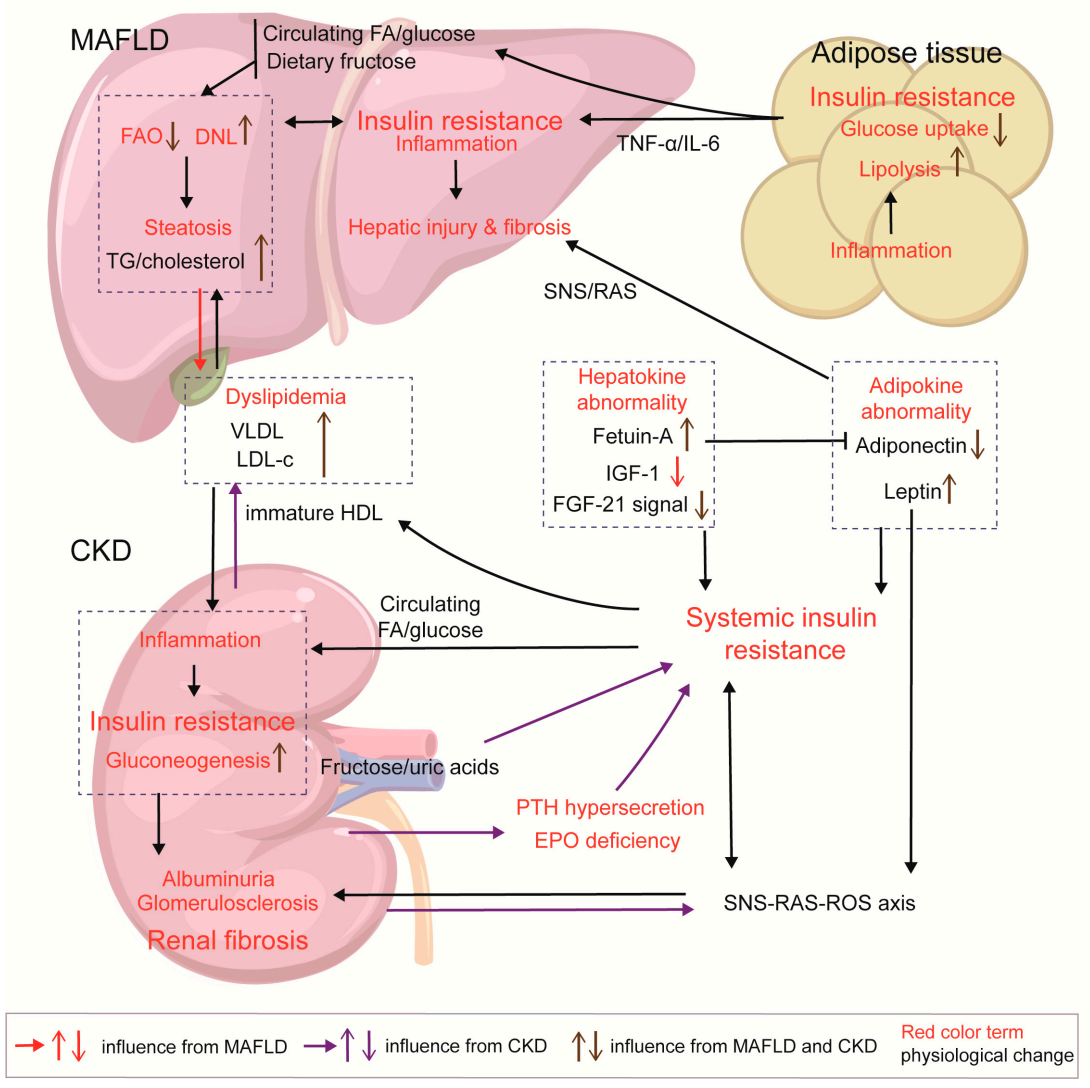
Insulin resistance in MAFLD and CKD and the interaction between the two diseases. In NAFLD, lipid overload triggers inflammatory responses and insulin resistance in adipocytes, acting as a pump for the export of FFA/glucose and inflammatory factors. Hepatic insulin resistance can be driven by high-fructose diets, dysregulation of FAO/DNL, and the formation of lipotoxic products, as well as by increased energy input from peripheral tissues. In CKD, HDL immaturity, increase in fructose/uric acid axis, decreased EPO production, and PTH hypersecretion contribute to systemic insulin resistance. CKD and diabetes can both cause HDL immaturity, leading to reduced HDL-c and increased LDL-c levels; meanwhile, MAFLD enhances VLDL output, exacerbating this pattern of dyslipidemia. Increased deposition of lipoproteins (VLDL and LDL-c), FAs, and glucose in the kidneys induces lipotoxicity and inflammatory response, which stimulate gluconeogenesis activation and insulin resistance in the kidneys; insulin resistance in the kidneys exacerbates glomerulosclerosis and renal fibrosis. MAFLD and CKD both exhibit abnormalities in adipokines and hepatokines, which induce systemic insulin resistance; abnormal adipokines cause damage to glomerulus and liver cells. Within the SNS/RAS/ROS axis, insulin, and adipokine, there is a feedback loop amplifying the interactions among them, and then leptin/SNS/RAS axis activates fibrotic signals in the liver and kidneys.

Insulin resistance in CKD also exacerbates MAFLD through the activation of SNS and RAS. In CKD, sympathetic reflexes from the kidneys and peripheral tissue stimulate the RAS/ROS axis to enhance insulin resistance in adipocytes and skeletal cells [153]. Adipocytes in inflammatory states can produce leptin, angiotensin II, and NE. Vitamin D deficiency in CKD affects the inhibition of RAS activation and release of angiotensin II [154]. Correspondingly, hyperleptinemia, increased angiotensin II levels, and hyperinsulinemia enhance renal SNS activation [117]. Though the liver serves as a nonclassical target organ for RAS and SNS, HSCs, Kupffer cells, and hepatocytes express AT-1 and the adrenoceptor. Angiotensin II/aldosterone stimulation has been found to promote NF-κβ signaling to mediate inflammation and hepatic fibrosis in rats [155]. Sigala et al. found that the adrenoceptor is up-regulated in human cirrhotic NAFLD, leading to HSC proliferation and collagen deposition upon SNS-neurotransmitter stimulation [156]. Another study found that the NE-adrenoceptor activated the release of TNF-α in a mouse model of liver cirrhosis [157]. This fibrotic process, which can also be induced through the angiotensin II/TGF-β1 pathway, is evident in the synergistic effect of insulin resistance, SNS, and RAS, which together aggravate liver injury (Figure 4).

### 5.4. The Effect of Fructose

Modern diets, such as SSBs, are rich in sucrose and high-fructose corn syrup. In MAFLD, Fructose metabolism proceeds ten-fold faster than glucose, generating excessive ROS and supplying G3P and acetyl-CoA for DNL [158]. Fructose is metabolized into fructose-1-phosphate by fructokinase, generating glycolytic intermediates that provide essential substrates, such as G3P and acetyl-CoA for lipogenesis. Perpetual fructose consumption promotes DNL through the activation of SREBP-1c/ChREBP downstream enzymes, such as ACC and FA synthase [159]. Up-regulation of CD36 induced by excess high fructose input mediates steatosis in MASLD [160]. In rats consuming a high fructose diet, hepatic activation of TLR4, NLRP3, NFκB, and JNK with the inhibition of AMPK leads to inflammation and insulin resistance [161]. Fructose also promotes hepatic fibrosis via oxidized coenzyme Q9 and nitro-oxidative stress [162].

In CKD, overconsumption of fructose escalates obesity, insulin resistance, and hyperuricemia [163]. Fructokinase converts fructose to fructose-1-phosphate, generating AMP for uric acid synthesis, while renal fructose catabolism supplies pyruvate for gluconeogenesis. Apart from dietary fructose, endogenous fructose production, enhanced by hyperglycemia and renal ischemia, further accelerates CKD progression [164]. In fructose-induced MetS, pro-inflammatory mediators such as uric acid and C-reactive protein are identified as drivers of renal injury [165]. Despite the previous belief that uric acid was not a significant contributor to metabolic problems, uric acid directly induces hepatic steatosis and insulin resistance through NLRP3 activation [166]. Thus, excessive fructose and uric acid accumulation represent convergent therapeutic targets in MAFLD and CKD.

## 6. Potential Therapies Targeting Metabolic Disorders in NAFLD and CKD

**Figure 5:**
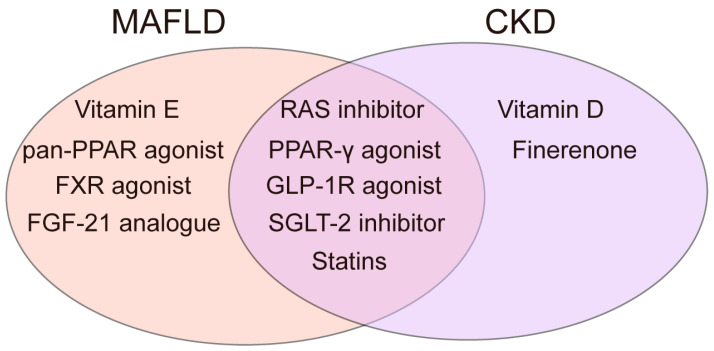
Drugs with verified clinical potential shared by two diseases.

To date, only resmetirom has been approved by the FDA in 2024 as a standard treatment for MAFLD. As a selective thyroid receptor agonist, resmetirom is indicated for alleviating fibrosis non-cirrhotic MASH and reducing hepatic fat accumulation [167]. However, several medications for obesity, diabetes, and cardiovascular diseases are recommended in MAFLD guidelines. For CKD, in addition to RAS inhibitors (RASIs) and SGLT-2 inhibitors (SGLT-2I) as first-line drugs, treatment also involves multiple complications, such as drugs to correct anemia, calcium supplements, antiplatelet agents, other antihypertensive drugs, antidiabetic drugs, etc. [168]. With the goal of correcting metabolic disorders, there is potential for overlap in the use of medications for MAFLD and CKD. Metabolism-related medications for MAFLD and CKD are listed in Table 1 and Table 2. Among them, the shared drugs used by two diseases with proven clinical potential are shown in Figure 5.

### 6.1. Vitamin

Active vitamin D, as a drug for the routine treatment of metabolic bone disease in CKD, can inhibit PTH hypersecretion, the release of inflammatory factors, and even RAS activation to alleviate albuminuria and insulin resistance [123]. The previous clinical study have confirmed that 1,25(OH)2D3 treatment can alleviate dyslipidemia and insulin resistance in patients with uremia [122]. Active VDRAs, such as calcitriol, have been shown to reduce homeostasis model assessment of insulin resistance (HOMO-IR) and liver function indices (ALT) in NAFLD patients deficient in 1,25(OH)2D3 [202]. Although short-term supplementation with Vitamin D and VDRA can alleviate insulin resistance, long-term use may cause abnormal blood calcium and phosphorus levels in MAFLD patients without deficiency of 1,25(OH)2D3.

Vitamin E ameliorates insulin resistance by down-regulating CD36 and up-regulating PPAR-γ-dependent adiponectin and improves oxidative stress by reducing ox-LDL and MDA production [169]. Guidelines suggest that in adults with NASH without diabetes, 800 IU/d rrr-α-tocopherol may be considered to improve inflammation, but it does not reverse fibrosis [170]. In adults with NASH without diabetes, 800 IU/d rrr-α-tocopherol may be considered. According to a 30-year follow-up trial of 4038 American adults, dietary vitamin E and tocopherol isoforms reduced the incidence of CKD [203]. For CKD, the therapeutic window for vitamin E is narrow, and excessive doses increase the risk of bleeding. The long-term safety of its use needs to be clinically verified.

### 6.2. Renin Angiotensin System Inhibitors and Mineralocorticoid Receptor Antagonist

Angiotensin-converting enzyme inhibitors (ACEIs) and angiotensin receptor blockers (ARBs), which belong to RASIs, are the first-line drugs for managing CKD hypertension, and they can alleviate metabolic problems. Huang et al. found that telmisartan, which is one of the ARB family and also partly functions as PPAR-γ agonist, improved insulin resistance by activating AMPK and diminishing ER stress and alleviated hepatic steatosis in mice fed a HFD [171]. In a randomized controlled trial (RCT) on MASH patients, telmisartan activated PPAR-γ and reduced blood TG and total cholesterol to alleviate the HOMO-IR, steatosis, inflammation, and hepatic fibrosis [172]. A nationwide database-based analysis published by *Hepatology* in 2025 showed that ACEI/ARB use significantly reduced the probability of death, CVD, and risk of hepatic events (e.g., ascites and hepatic encephalopathy) in MASLD [173]. Telmisartan was found to ameliorate vascular dysfunction and fibrosis by activating PPAR-γ and blocking AT-1 receptors in CKD model of 5/6 nephrectomized rats with hypertension [188]. A meta-analysis of 18 RCTs including 1739 participants with a mean eGFR of 22.2 mL/min/1.73 m^2^ showed that the use of RASIs could delay progression to ESRD with replacement therapy [189]. Importantly, the dilating effect on the efferent arteries and hyperkalemia induced by RASIs is noticeable. When using RAS blockers, especially in patients with CKD, electrolyte and eGFR decreases should be monitored, and dose adjustments should be made promptly; neither discontinuation nor dual use of ARB and ACEI is recommended [168].

A non-steroidal mineralocorticoid receptor antagonist (MRA), such as finerenone, is recommended to be added to a RASI and a SGLT-2I for treatment of CKD with T2D in Kidney Disease: Improving Global Outcomes (KDIGO) guidelines. Finerenone antagonizes the action of aldosterone, down-regulates inflammatory fibrosis genes such as TGF-β1 and collagen 1A1, reduces water and sodium retention, and has excellent renal and cardiac protective effects in diabetic nephropathy [190]. For the liver, there is only one small sample size RCT involving 48 MASH patients, which showed improvement in fibrosis after MRA treatment and benign liver safety [204]. However, research on MRA treatment for MAFLD remains limited.

### 6.3. Insulin-Sensitizing Agents

As an insulin-sensitizing agent, thiazolidinedione (TZD) activates PPAR-γ and up-regulates adiponectin levels in adipose tissue, stimulating AMPK and PPAR-α and enhancing FAO and glucose uptake [205]. The 2023 American Association for the Study of Liver Diseases (AASLD) practice guidelines indicate that pioglitazone can be considered in cases of MAFLD combined with T2D, but caution should be exercised regarding side effects [170]. In patients with NASHs, one of the TZD family, pioglitazone, improved insulin resistance, significantly reduced intrahepatic fat, and attenuated histological changes and hepatic fibrosis [174]. An RCT of diabetes patients showed that pioglitazone lowered both hemoglobin A1c and fetuin-A levels [175]. Sun et al. found that pioglitazone ameliorated mitochondrial dysfunction by inhibiting ROS production and protected the kidneys from fibrotic progression by reducing TGF-β1 signaling and fibronectin and collagen I levels in a CKD model of 5/6 nephrectomized rats [191]. A meta-analysis showed that TZD reduced microalbuminuria by an average of 25–30%, slowed the annual rate of decline in eGFR by about 1 mL/min/year, but increased hospitalization for heart failure [192]. Recognition of the adverse effects of TZD, which include weight gain, water–sodium retention, edema, and the possibility of developing heart failure and severe renal impairment, should be maintained. The American Diabetes Association (ADA)-KDIGO consensus report recommends that TZDs may be considered when first-line medications are not tolerated; however, they should be used with caution in patients who are overweight or at risk of heart failure.

In recent years, lanifibranor (pan-PPAR agonist) and saroglitazar (dual PPAR-γ/α agonist) presented lower degrees of edema, weight gain, and incidence of heart failure. Lanifibranor reversed or attenuated fibrosis in MASH in clinical phase II trials and improved TG, HDL-c, HOMA-IR, and other biochemical abnormalities, demonstrating superior anti-fibrotic and anti-inflammatory effects [176]. Saroglitazar has been shown to be pharmacokinetically stable in severe renal injury and cholestatic liver disease, with no demonstrated safety concerns [2]. The safer, more comprehensive effect makes the new PPAR agonist drug more feasible for concurrent use in MAFLD and CKD.

### 6.4. Glucagon-like Peptide 1 Receptor Agonists

Glucagon-like peptide-1 (GLP-1) is an incretin secreted by distal-ileal L-cells that augments glucose-stimulated insulin release. In T2D, GLP-1R agonists (GLP-1RA) improve obesity-related insulin resistance by enhancing β-cell insulin secretion and suppressing hepatic DNL [206]. The US FDA has approved once-weekly semaglutide for chronic weight management in adults with obesity or those who are overweight, providing a therapeutic rationale for MAFLD. GLP-1RA therapy lowers circulating fetuin-A concentrations and mediates the GLP-1/FGF-21 axis in clinical studies of MASLD [63]. A meta-analysis of RCTs demonstrated that liraglutide, semaglutide, and exenatide significantly reduced hepatic fat content and improved the histological manifestation of MASH compared with a placebo [177]. In a phase III study of MASH, semaglutide also decreased hepatic fibrosis, attenuated lobular inflammation, and resulted in marked weight loss [178]. In the kidneys, GLP-1 down-regulates epithelial sodium channel expression in the proximal tubules, thereby reducing sodium reabsorption and intraglomerular pressure [207]. In an HFD-induced CKD rat model, liraglutide lessened renal lipid accumulation, activated the Sirt1/AMPK/PGC-1α axis, and preserved mitochondrial function [193]. Li et al. reported that liraglutide inhibited the ECM secretion of PTECs treated with TGF-β1 and ameliorated renal fibrosis in CKD mice with unilateral ureteral obstruction (UUO) [194]. Clinically, once-weekly dulaglutide (1.5 mg) lowered the composite risk of ≥40% eGFR decline or progression to ESRD in patients with T2D and CKD [195]. Drugs administered at low eGFR increase the likelihood of intractable gastrointestinal reactions and need prompt adjustment; Liraglutide along with exenatide is partially excreted by the kidneys, administration is recommended with eGFR ≥ 15 mL/min/1.73 m^2^ for liraglutide and eGFR ≥ 60 mL/min/1.73 m^2^ for exenatide [208]. GLP-1RA has become one of the few “same-target, dual-action” drugs that link MAFLD and CKD by integrating metabolic–inflammatory–hemodynamic signaling through multiple pathways.

### 6.5. Sodium–Glucose Cotransporter 2 Inhibitors

SGLT-2I diminish the reabsorption of glucose and sodium in the proximal tubules, resulting in decreased albuminuria, water–sodium retention, RAS activation, and blood glucose levels [196]. Dapagliflozin and empagliflozin, the SGLT-2I, have been approved by the FDA as anti-diabetic drugs and are officially listed as first-line drugs for CKD in KDIGO’s 2024 guidelines. It used to be thought that SGLT-2I was not recommended CKD patients with eGFR < 45 mL/min/1.73 m^2^; a recent study showed that CKD patients given dapagliflozin treatment with eGFR < 30 mL/min/1.73 m^2^, compared to a placebo, similarly demonstrated decelerated decreases in eGFR, with reduced rates of mortality and cardiovascular events, which is consistent with the effects observed in CKD patients with eGFR between 30 and 70 mL/min/1.73 m^2^ [198]. Dapagliflozin attenuates renal fibrosis manifestations and ECM deposition in diabetic mice via angiotensin II/TGF-β1 signaling [196]. In HFD-induced obese mice, empagliflozin also inhibited CD36 expression in PTECs through the PPARγ pathway, reducing the deposition of lipotoxic products and cell apoptosis [197]. In the liver of T2D mice with HDF treatment, the animal and in vitro experiments documented that dapagliflozin regulated the AMPK/mTOR pathway and inhibited DNL-related enzyme ACC, improving hepatic TG accumulation and steatosis [179]. Empagliflozin is reported to alleviate inflammation and steatosis in MASH mice with improvements in HOMA-IR, inflammatory factors, and fibrosis markers [180]. A 2025 RCT found that dagliflozin inhibited the progression of hepatic fibrosis in MASH without significantly altering histology [181]. The effects of SGLT-2I on the kidney are relatively well defined, and institutions have initiated studies on the combination of SGLT-2I and lanifibranor in MAFLD/T2D to reduce edema through hemodynamic improvement and to efficiently reverse hepatic fibrosis and systemic inflammation. The ADA has included eGFR ≥ 20 as a routinely recommended threshold, and more clinical evidence is needed on the safety of pre-dialysis CKD (eGFR < 15); the risk of diabetic ketoacidosis and urinary tract infection while co-administering insulin need to be cautioned.

### 6.6. Farnesoid X Receptor Agonists

The farnesoid X receptor (FXR) in hepatocytes, PTECs, and ileal epithelium regulates the homeostasis of bile acids, lipids, and glucose. The FXR agonist enhances CPT-1-mediated FAO, inhibits DNL activity, induces ileal and hepatic production of FGF-19 to inhibit bile acid overproduction, and lowers the levels of hepatic lipids to reduce VLDL output. In a randomized double-blind trial of patients with T2D and NAFLD, the group treated with obeticholic acid, a natural agonist of FXR, exhibited significantly lower AST and ALT values, increased insulin sensitivity, and improved hepatic fibrosis compared to a placebo [182]. In the phase 2a/b trial of a new-generation FXR agonist, tropifexor, NASH fibrotic signaling and hepatic fat receded significantly compared to a placebo, and no pharmacological hepatotoxicity was observed [183]. The FXR agonist attenuated lipid accumulation, lipotoxicity, and ox-LDL/β-catenin-mediated activation of the fibrotic pathway in renal tubules in an HFD mice model [76]. Major adverse effects of FXR agonists, including pruritus, hepatotoxicity, and LDL-c burden due to cholesterol reflux, warrant particular caution. FXR is a common target in the liver and kidneys through the lipo-inflammatory–fibrotic axis, but longer-term, larger clinical trials are needed to validate its safety profile and the application in CKD.

### 6.7. FGF-21 Analogue

A recent meta-analysis of nine IIb/IIa RCTs, including 1054 MASH patients, showed that the use of FGF-21 analogues significantly improved hepatic fibrosis; it also significantly improved liver fat content, transaminases, and dyslipidemia indices (TG, HDL-C, and LDL-C) [184]. Mendelian randomization studies using missense variants that mimic pharmacological activation of FGF-21 further show associations with higher eGFR and a reduced risk of CKD [209]. Although related trials are sparse in CKD and renal outcomes remain undefined, the genetics analysis and safety data provide a strong rationale for ongoing and future clinical studies.

### 6.8. Statins

Inhibitors of 3-hydroxy-3-methylglutaryl-coenzyme A (HMG-CoA) reductase and ligand, known as statins, reduce levels of TG, total cholesterol, and LDL-c. In a NASH model, statins treatment upregulated hepatic PPAR-α and CPT-1α, thereby augmenting mitochondrial FAO [185]. In another NAFLD model, simvastatin mitigated steatosis, fibrosis and inflammatory signaling by suppressing oxidative and ALE-RAGE stress [186]. Clinically, a RCT in 32 patients with metabolic syndrome and moderate-to-severe MAFLD showed that hepatic lipid content declined in parallel with decreases in LDL-C, ApoB, and FFA [187]. Consistent with these findings, the AASLD practice guidance endorses statins as safe in MAFLD and advises early initiation to lessen cardiovascular risk [170]. Renal data are similarly encouraging. In 5/6 nephroectomized rats, after atorvastatin reduced LDL-C levels, cholesterol crystals were significantly reduced, NRLP3 activation and foam cell infiltration were alleviated, and renal aquaporin-2 AQP2 expression and renal function were improved [199]. Rosuvastatin has been found to alleviate albuminuria and inflammation and reduce urinary albumin–creatinine ratio in CKD patients [200]. High-dose atorvastatin (≥40 mg) and rosuvastatin (≥20 mg) significantly reduced the progression of eGFR in CKD patients [201]. Statin therapies have slight risk of myotoxicity, necessitating dosage adjustments by severity of renal impairment, but discontinuation is considered only when transaminases exceed three times the upper limit of normal [210].

## 7. Conclusions

Our review highlights that metabolic dysfunction-associated fatty liver disease (MAFLD) is a potent initiator and accelerator of chronic kidney disease (CKD), whereas CKD can reciprocally worsen hepatic steatosis. On the one hand, excessive ectopic lipid accumulation and insulin resistance unify MAFLD and CKD pathogenesis, promoting their oxidative stress, inflammation, and fibrosis through shared pathways, such as dyslipidemia induced by the impairment of related enzymes and receptors, activation of inflammatory signals mediated by cell senescence, ALEs, ox-LDL, CD36, and RAGE/TLR-4, and insulin signaling impairment driven by PPAR signals and LCFA/DAG/ceramide. Systemic involvement via the SNS/RAS/ROS axis, exacerbated by insulin resistance and altered adipokine/hepatokine profiles, intensifies mutual hepatic and renal injury. On the other hand, advanced CKD results in excessive lipotoxins, uremic toxins, and PTH, as well as deficiency in EPO, in a unique pattern that causes systemic inflammation and insulin resistance. Hepatic insulin resistance is selective, as evidenced by preserved gluconeogenesis and DNL effects but impaired glucose uptake and glycogen synthesis, whereas peripheral insulin resistance is biased toward enhanced lipolysis. The interaction between MAFLD and CKD reinforces the loop of “extrahepatic energy donation and hepatic storage”.

Emerging pharmacotherapies, including statins, PPAR-γ agonists, GLP-1RAs, RASIs, and SGLT-2I, have demonstrated multi-target metabolic effects and clinical efficacy in both MAFLD and CKD. Among these agents, GLP-1RAs and SGLT-2I are becoming first-line therapies in MAFLD-CKD comorbidity, owing to their combined metabolic and hemodynamic benefits, alongside accumulating evidence supporting their long-term safety. FXR agonists, FGF-21 analogues, and pan-PPAR agonists have so far only been validated in MAFLD but possess therapeutic targets abundantly expressed in multiple organs, including the liver and kidney, and exhibit good tolerability with manageable side effects. Future clinical studies should focus on FXR agonists with minimal hepatotoxicity and on dedicated CKD trials for FGF-21 and pan-PPAR agonists. The combination of pan-PPAR agonists and SGLT-2I holds particular promise due to the relief of blood volume burden and insulin resistance. Finally, although selective CD36 inhibitors have not yet been developed and entered clinical use, their research is warranted given CD36’s central role in mediating metabolic crosstalk along the liver–kidney axis.

## Figures and Tables

**Table 1:** Drugs that intervene in the metabolism of MAFLD.

Type	Object	Mechanism and Influence on the Liver	Reference
Vitamin E	NASH adults without T2D	Down-regulate CD36, up-regulate PPAR-γ/adiponectin, and reduce ox-LDL and MDA levels; hepatic inflammation↓ and incidence of CKD↓	[169,170]
RAS inhibitors	Obese mice with HDF	Activate PPAR-γ/AMPK, inhibit RAS, and improve insulin resistance; steatosis↓	[171]
	MASH patients and MASLD patients	Activate PPAR-γ, and reduce TG and cholesterol levels; HOMO-IR↓, inflammation↓, steatosis↓, hepatic fibrosis↓, and morbidity↓	[172,173]
PPAR-γ agonists	NASH patients and diabetes patients	Activate adiponectin/AMPK/PPAR-α, inhibit Fetuin-A, and enhance FAO; intrahepatic lipid↓ and hepatic fibrosis↓	[174,175]
Pan-PPAR agonists	MASH patients	Improve TG and HDL levels; HOMA-IR↓, inflammation↓, and hepatic fibrosis↓	[176]
GLP-1R agonists	MASLD/MASH patients	Inhibit Fetuin-A and induce FGF-21; hepatic fat content↓, inflammation↓, and hepatic fibrosis↓	[63,177,178]
SGLT-2 inhibitors	T2D mice with HDF and MASH mice	Modulate AMPK/mTOR and inhibit ACC; inflammation↓, hepatic TG accumulation↓, HOMA-IR↓, and hepatic fibrosis↓	[179,180]
	MASH patients	Hepatic fibrosis↓	[181]
FXR agonists	NAFLD patients with T2D and NASH patients	Enhance CPT-1/FAO, inhibit DNL, improve insulin sensitivity, and reduce VLDL; intrahepatic lipids↓, transaminase↓, and hepatic fibrosis↓	[182,183]
FGF-21 analogue	MASH patients	Activate PGC-1α, inhibit SREBP-1c, and improve insulin sensitivity; hepatic fat content↓, transaminase↓, and hepatic fibrosis↓	[184]
Statins	NAFLD/NASH mice	Activate PPAR-α/CPT-1α/FAO and reduce ALE-RAGE signals; inflammation↓, steatosis↓, and hepatic fibrosis↓	[185,186]
	MAFLD patients	Reduce dyslipidemia indices (LDL-c, Apo B, FFA, TG, and total cholesterol); hepatic fat content↓ and incidence of CVD↓	[170,187]

The arrow↓, alleviation of condition, or decrease of negative indicators; the arrow↑, enhancement of function.

**Table 2:** Drugs that intervene in the metabolism of CKD.

Type	Object	Mechanism and Influence on the Kidney	Reference
Vitamin D	CKD/uremia patients	Inhibit PTH hypersecretion, inhibits RAS, and improve dyslipidemia and insulin resistance; albuminuria↓	[122,123]
RAS inhibitors	5/6 nephrectomized rats	Activate PPAR-γ and inhibit RAS; renal fibrosis↓	[188]
	CKD patients	Incidence of ESRD↓	[189]
Finerenone	Diabetic nephropathy	Antagonize aldosterone and maintain water-sodium balance; renal fibrosis↓	[190]
PPAR-γ agonists	5/6 nephrectomized rats	Inhibit TGF-β1 and collagen I; renal fibrosis↓	[191]
	CVD patients	Microalbuminuria↓; eGFR decline↓	[192]
GLP-1R agonists	HFD-induced CKD rats and UUO rats	Reduce sodium reabsorption, activate Sirt1/AMPK/PGC-1α, and inhibit TGF-β1 and ECM secretion; renal fat accumulation↓; renal fibrosis↓	[193,194]
	CKD patients with T2D	Incidence of ESRD↓; eGFR decline↓	[195]
SGLT-2 inhibitors	Obese/diabetic mice	Inhibit RAS/TGF-β1/ECM secretion, inhibit the absorption of glucose and sodium, and inhibit CD36 in PTECs; albuminuria↓, renal fibrosis↓, and renal lipotoxicity↓	[196,197]
	CKD patients	eGFR decline↓ and morbidity↓.	[198]
Statins	5/6 nephroectomized rats	Reduce dyslipidemia indices and alleviate NRLP3 activation; inflammation and foam cell infiltration↓, dyslipidemia↓, and renal function↑	[199]
	CKD patients	Albumin/creatinine ratio↓, inflammation↓, and eGFR decline↓	[200,201]

The arrow↓, alleviation of condition, or decrease of negative indicators; the arrow↑, enhancement of function.

## Data Availability

Data availability is not applicable to this article as no new data were created or analyzed in this study.
